# Phytochemical Composition, Antioxidant and Anti-Inflammatory Activities, and Protective Effect Against LPS-Induced Liver Injury in Mice of *Gerbera delavayi* Franch

**DOI:** 10.3390/antiox15010143

**Published:** 2026-01-22

**Authors:** Hongmei Yin, Yinrong Zhao, Rouxian Hu, Jing Yang, Yuanhang Chen, Huaqiao Tang, Xiaoyan Li, Gang Ye, Fei Shi, Cheng Lv, Ling Zhao

**Affiliations:** 1 College of Animal Science, Xichang University, Xichang 615000, China; xcc20180083@xcc.edu.cn (H.Y.); xcc04000032@xcc.edu.cn (X.L.); 2 College of Veterinary Medicine, Sichuan Agricultural University, Chengdu 611130, China; zhaoyinrong@stu.sicau.edu.cn (Y.Z.); hurouxian@stu.sicau.edu.cn (R.H.); yangjing4@stu.sicau.edu.cn (J.Y.); 2023403203@stu.sicau.edu.cn (Y.C.); huaqiao_tang@sicau.edu.cn (H.T.); 13521@sicau.edu.cn (G.Y.); fei_shi@sicau.edu.cn (F.S.); l3414@sicau.edu.cn (C.L.)

**Keywords:** *Gerbera delavayi* Franch, LC-MS, antioxidant, anti-inflammatory, acute liver injury, macrophages, RAW264.7 cells, Nitric oxide

## Abstract

The main objective of this study was to preliminarily analyze the major flavonoid and phenolic acid components of the ethanolic extract of *Gerbera delavayi* Franch (E-GDF), and to evaluate its anti-inflammatory and antioxidant properties in lipopolysaccharide (LPS)-stimulated murine macrophage RAW264.7 cells and systemic inflammation mouse models. Results indicated that E-GDF was rich in flavonoids (16.35 ± 0.19 mg RT/g d.w. Plant Material) and polyphenolic compounds (36.15 ± 0.20 mg GAE/g d.w. Plant Material). LC-MS analysis of E-GDF revealed that its major flavonoid components included kaempferol glycosides, luteolin, and their glycosylated derivatives, while its phenolic acids were predominantly chlorogenic acid, caffeic acid, ferulic acid, and their corresponding glycosides. E-GDF exhibited good antioxidant activities, including the scavenging of DPPH, ABTS, ^•^OH, and O_2_^•−^ radicals. E-GDF treatment significantly inhibited the production of ROS and inflammatory mediators (NO, IL-6, TNF-α) in LPS-stimulated macrophages (RAW 264.7), while concurrently down-regulating the mRNA expression of COX-2, IL-1β, Casp1, and GSDMD-1. In addition, in vivo experiments revealed that E-GDF treatment effectively reduced the serum LPS, AST levels, as well as hepatic TNF-α, IL-6 levels in mice with LPS-induced acute liver injury. Furthermore, E-GDF significantly ameliorated LPS-induced liver pathological damage. These results provide a basis for *G. delavayi* as a potential antioxidant, anti-inflammatory, and hepatoprotective herbal medicine.

## 1. Introduction

Inflammation is a complex physiological and pathological response of vascularized tissues to endogenous or exogenous injurious stimuli. Its fundamental pathological alterations include degeneration, exudation, and hyperplasia of local tissues, which are clinically manifested by the classic signs of redness, swelling, heat, and pain [1]. Inflammation can be initiated by diverse factors, such as pathogens, irritants, and damaged cells [2]. Lipopolysaccharide (LPS), a component of the outer membrane of Gram-negative bacteria, is a potent immunostimulant. Upon host infection, LPS is released following bacterial disintegration and elicits broad inflammatory responses. Once introduced into the body, LPS stimulates multiple cell types, including neutrophils, macrophages, and vascular endothelial cells, to synthesize and release various inflammatory cytokines. Key factors such as IL-1β, IL-6, and TNF-α play central roles in this process. These mediators promote increased vascular permeability, fluid exudation, and lymphocyte migration to inflammatory sites, ultimately manifesting as systemic immune stress.

The liver is a vital metabolic organ in the human body. Liver injury can be caused by various factors, including viruses, alcohol, and endotoxins [3], and LPS can further exacerbate the degree of liver injury by activating a variety of inflammatory pathways [4]. In recent years, it has been found that oxidative stress and inflammation are key drivers in the onset and progression of liver diseases [5]. During oxidative stress injury, cells release inflammatory mediators such as IL-6, IL-1β, and TNF-α, further exacerbating liver injury [6].

Macrophages are crucial innate immune cells that are widely distributed throughout various organs and tissues. They play a key role in host defense and are involved in numerous pathophysiological processes [7]. The LPS-induced inflammatory response in murine macrophage RAW264.7 cells is a widely used in vitro model for studying the anti-inflammatory activities of plant extracts [8].

*Gerbera delavayi* Franch (*G. delavayi*, GDF), is a plant belonging to the genus Gerbera (family Asteraceae). It primarily grows in the Hengduan Mountains region, specifically in southern Yunnan Province and Sichuan Province, China. GDF is used in Yi folk medicine to treat ailments such as abdominal pain and asthma. As a type of ethnomedicinal plant primarily employed in the Yi-populated regions of China, GDF has received limited scientific attention to date [9]. Previous investigations have demonstrated that members of the Asteraceae family are rich in coumarins, sesquiterpene lactones, and other bioactive constituents [10,11,12] and exhibit broad pharmacological activities, including anti-inflammatory [13], anti-proliferative [14], anti-asthmatic [15], anti-obesity [16], and vasorelaxant effects [17]. These findings provide a compelling scientific rationale for the in-depth study of Gerbera species.

In the present study, the flavonoids and phenolic acids in the *G*. *delavayi* extract were systematically profiled via LC–MS. Furthermore, the antioxidant and anti-inflammatory activities of the extract and its hepatoprotective effect against LPS-induced acute liver injury in mice were comprehensively evaluated through both in vitro and in vivo experiments.

## 2. Materials and Methods

### 2.1. Materials and Reagents

All chemical reagents and assay kits used in this study were obtained from commercial suppliers.

Specifically, Gallic acid, Folin-Phenol, Rutin, Aluminum trichloride, 1,1-diphenyl-2-trinitrophenylhydrazine (DPPH), 2,2-biazobis (3-ethyl-benzothiazole-6-sulfonic acid) diammonium salt (ABTS), and Potassium persulfate were purchased from Genye Biologicals (Suzhou, China). DMEM medium was acquired from Gibco (Grand Island, NY, USA). LPS (*Escherichia coli* serotype O55:B5) was obtained from Sigma (St. Louis, MO, USA). Fetal bovine serum (FBS) was sourced from Sijiqing (Hangzhou, China). Anhydrous ethanol, anhydrous sodium carbonate, and Dimethyl sulfoxide (DMSO) were procured from Cologne Chemical (Chengdu, China). Ultra-purified water was obtained using a Milli-Q Synthesis System (Millipore, Burlington, MA, USA).The All-in-one 1st Strand cDNA Synthesis SuperMix (with gDNA Purge) was supplied from Toyobo (Osaka, Japan)). Mouse IL-6 and TNF-α ELISA kits were provided by RuiXin Biotech (Guangzhou, China). Nitric oxide (NO), alanine aminotransferase (ALT), and aspartate aminotransferase (AST) assay kits were purchased from Nanjing Jiancheng Bioengineering Institute (Nanjing, China). Cell Counting Kit-8 (CCK-8), BCA protein assay kit, and RIPA lysis buffer were obtained from Beyotime Biotechnology (Shanghai, China). TRIzol reagent was procured from Omega Bio-Tek (Norcross, GA, USA).

### 2.2. Preparation of Ethanolic Extract of Gerbera delavayi Franch (E-GDF)

*G. delavayi* was collected in Xichang City, Sichuan Province, China [28°07′05.9127″ N, 102°17′08.0247″ E (GCJ-02 datum), 2012 m a.s.l.], and identified by Associate Professor Lixia Li, a taxonomist who has specialized in medicinal-plant identification for more than ten years. Following the protocol of He et al. for *Gerbera anandria*, 75% ethanol was employed as the solvent [18]. The shade-dried plant material was pulverized and 200 g of the powder was extracted with 10 BV 75% ethanol under reflux for 1 h for three times. The combined filtrates were concentrated under reduced pressure to yield an extract of 12.2 g. The extract was freeze-dried and retained at −20 °C until use. Working solutions were prepared immediately before use.

### 2.3. Determination of Total Polyphenol Content and Total Flavonoid Content

#### 2.3.1. Total Polyphenol Content

The total polyphenol content (TPC) in E-GDF was determined using the Folin–Ciocalteu method with gallic acid equivalents (GAE) as the standard [19]. A standard curve was constructed by adding aliquots (0–1.5 mL) of gallic acid solution (500 μg/mL) to test tubes, followed by the addition of 1.0 mL Folin–Ciocalteu reagent and 2.0 mL of 12% sodium carbonate solution. The mixture was diluted to 4 mL with deionized water, incubated at 25 °C for 2 h in the dark, and the absorbance was measured at 760 nm against a blank. For sample analysis, 200 mg E-GDF/mL was processed identically. The TPC was calculated based on the measured absorbance and the standard curve.

#### 2.3.2. Total Flavonoid Content

The total flavonoid content (TFC) in E-GDF was determined using the colorimetric method with rutin (RT) as the standard [20]. A standard curve was prepared by adding aliquots (0.1–1.0 mL) of RT solution (0.3 mg/mL) to 10 mL volumetric flasks, followed by the addition of 1.0 mL of 60% ethanol, 0.5 mL of 5% sodium nitrite, 0.5 mL of 10% aluminum nitrate, and 4.0 mL of 4% sodium hydroxide. After standing for 15 min, absorbance was measured at 510 nm. For sample analysis, 200 mg E-GDF/mL was processed identically, and TFC was calculated based on the standard curve.

#### 2.3.3. Detection of Non-Targeted Metabolites of E-GDF in Positive and Negative Ion Mode by the LC-MS Method

Liquid chromatography-mass spectrometry (LC-MS) was employed to quantify and simultaneously analyze the flavonoid and phenolic acid constituents of E-GDF. Briefly, 100 μL of E-GDF was aliquoted into a 1.5 mL EP tube, mixed with 300 μL of 95% methanol (LC-MS Grade, Sigma-Aldrich, St. Louis, MO, USA), vortexed for 30 s, and centrifuged at 17,000 rpm and 20 °C for 10 min. The supernatant was transferred to an injection vial for LC-MS analysis. Chromatographic separation was performed on a Waters ACQUITY UPLC HSS T3 column (100 mm × 2.1 mm, 1.8 μm, Waters Corporation,  Milford, MA, USA) maintained at 40 °C. The mobile phase consisted of 0.1% formic acid in water (A) and 0.1% formic acid in acetonitrile (B). The gradient elution program was set at a flow rate of 0.4 mL·min^−1^ as follows: 5–10% B (0–2.5 min), 10–40% B (2.5–14 min), 40–95% B (14–24 min), 95% B (24–27 min), 95–5% B (27–27.1 min), and 5% B (27.1–30 min). The injection volume was 4 μL. Mass spectrometric detection was conducted using an AB Sciex TripleTOF 5600 system with an electrospray ionization (ESI) source operating in positive and negative ion modes with MSE continuous acquisition. Primary and secondary spectra were acquired based on the information-dependent acquisition (IDA) mode. Each cycle collected the top 15 precursor ions exceeding an intensity threshold of 100, within a mass range of 50–1200 Da. Collision energy was set to 30 eV. ESI parameters were as follows: nebulizing gas (GS1), 60 psi; auxiliary gas, 60 psi; curtain gas, 35 psi; temperature, 550 °C; ion spray voltage, 5500 V (positive) or –4500 V (negative).

### 2.4. Antioxidant Activity Assays

#### 2.4.1. DPPH Radical Scavenging Activity

The DPPH radical scavenging assay was performed according to a published protocol with minor modifications [21]. Briefly, 50 μL of E-GDF (20 mg/mL) was mixed with 150 μL of DPPH solution (0.08 mg/mL in anhydrous ethanol). A control group (50 μL of E-GDF with 150 μL anhydrous ethanol) and a negative control (50 μL anhydrous ethanol with 150 μL DPPH solution) were also prepared. All mixtures were incubated at room temperature in the dark for 30 min. Ascorbic acid at an equivalent concentration served as the positive control and was processed similarly. Absorbance was measured at 517 nm. The DPPH scavenging activity was calculated using the following formula:

(1) $$DPPH\;scavenging\;activity\left(\%\right)=1-\frac{A1-A2}{A0-A2}\times 100\%$$

where A1 is the absorbance of the sample, A2 is the absorbance of the control group, and A0 is the absorbance of the negative control group.

#### 2.4.2. ABTS Radical Scavenging Activity

The ABTS radical scavenging assay was conducted according to a published protocol with minor modifications [21]. The ABTS^+^ working solution was prepared by mixing 10 mL of 7 mM ABTS solution with 10 mL of 2.45 mM potassium persulfate, followed by reaction in the dark at room temperature for 6 h. The solution was then diluted approximately 55-fold with anhydrous ethanol to achieve an absorbance of 0.70 ± 0.02 at 734 nm. For the assay, 20 μL of E-GDF (20 mg/mL) was mixed with 150 μL of ABTS^+^ solution as the test group. The control group contained 20 μL of E-GDF and 150 μL anhydrous ethanol, and the negative control consisted of 20 μL anhydrous ethanol and 150 μL ABTS^+^ solution. Ascorbic acid at the same concentration was used as the positive control and processed identically. After 6 min of incubation, absorbance was measured at 734 nm. The ABTS radical scavenging activity was calculated using the following formula:

(2) $$ABTS\;scavenging\;activity\left(\%\right)=1-\frac{A1-A2}{A0-A2}\times 100\%$$

where A1 is the absorbance of the treatment group, A2 is the absorbance of the control group, and A0 is the absorbance of the negative control group.

#### 2.4.3. Hydroxyl Radicals Scavenging Activity

The hydroxyl radical scavenging capacity of E-GDF was evaluated according to a published protocol with minor modifications [22]. Briefly, 1 mL of E-GDF (20 mg/mL) was mixed with 1 mL of 9 mmol/L FeSO_4_, 1 mL of 9 mmol/L salicylic acid in ethanol, and 1 mL of 8.8 mmol/L H_2_O_2_. The mixture was incubated at 37 °C for 30 min, and absorbance was measured at 510 nm. Ascorbic acid at the same concentration served as the positive control. The hydroxyl radical scavenging activity was calculated using the following formula:

(3) $$hydroxyl\;radical\;scavenging\;activity\left(\%\right)=1-\frac{Ax-Ax1}{Ax0}\times 100\%$$

where Ax0 is the absorbance of the mixture (without sample), Ax is the absorbance of the sample, and Ax1 is the absorbance of the mixture (without sample and H_2_O_2_)

#### 2.4.4. Superoxide Radicals Scavenging Activity

The superoxide radical scavenging activity was assessed as follows [23]: 1 mL of E-GDF (20 mg/mL) was mixed with 2.0 mL of 0.1 mol/L Tris-HCl buffer (pH 8.2) and 0.4 mL of 6.0 mmol/L pyrogallol. After incubation at 25 °C for 5 min, the reaction was terminated by adding 0.8 mL of 10 mmol/L HCl. Absorbance was measured at 420 nm. Ascorbic acid at the same concentration served as the positive control. The scavenging activity was calculated using the following formula:

(4) $$superoxide\;radicals\;scavenging\;activity\;(\%)=1-\frac{Ax-Ax1}{Ax0}\times 100\%$$

where Ax0 is the absorbance of the mixture (without sample), Ax is the absorbance of the sample, and Ax1 is the absorbance of the mixture (without sample and pyrogallol)

### 2.5. In Vitro Cellular Experiments

#### 2.5.1. Cell Culture

The stock solution (0.2 g/mL crude drug) was diluted with distilled water to obtain working concentrations of 10, 50, 100, 200, and 400 μg/mL. All solutions were filtered through 0.22 μm micropore filters for sterilization and stored at −20 °C for future use.

Mouse RAW264.7 macrophages (ATCC: TIB-71; Typical Culture Collection Committee Cell Bank, Chinese Academy of Sciences) were cultured in DMEM medium supplemented with 10% fetal bovine serum (FBS) at 37 °C in a 5% CO_2_ humidified incubator.

#### 2.5.2. Cell Viability Assay

RAW264.7 cells in the logarithmic growth phase (100 µL) were seeded into 96-well plates at 1 × 10^4^ per well and allowed to adhere. The following groups were established, each with six replicate wells: a blank control group, a vehicle group, and 5 E-GDF treatment groups (final concentrations: 10, 50, 100, 200, and 400 μg/mL). After cell attachment, the medium was replaced with 100 µL of DMEM for the blank control, 100 µL of 0.1% DMSO for the vehicle group, or 100 µL of the corresponding concentration of E-GDF for the treatment groups. Following 8 h of incubation, 100 μL of CCK-8 reagent was added to each well, and the plates were further incubated for another 2 h. Absorbance was measured at 450 nm, and the cell viability of each group was calculated as follows:

(5) $$cell\;viability\;(\%)=\frac{\left(A1-A0\right)}{\left(A2-A0\right)}\times 100\%$$

where A0 is the absorbance of the blank control group, A1 is the absorbance of the E-GDF group, and A2 is the absorbance of the vehicle group.

#### 2.5.3. Nitric Oxide (NO) Assay

The grouping and treatments in the NO assay were identical to those described previously (2.5.2). After the cell adherence to the wall, the medium was replaced with 100 µL of DMEM for both the blank control and the model group, and 100 µL of the viral concentration of E-GDF in the E-GDF groups. After an additional 2 h incubation, the blank control group received additional DMEM, while the model and E-GDF groups were stimulated with LPS. All groups were then incubated for another 24 h. Cell culture supernatants were collected, and NO content was measured according to the manufacturer’s kit instructions.

#### 2.5.4. Reactive Oxygen Species (ROS) Assessment

Cell grouping and experimental treatments were performed as outlined in Section 2.5.2. Following the treatment period, cell culture supernatants were collected, and intracellular ROS levels were detected using flow cytometry. The proportion of ROS-positive cells in the control group was normalized to 100%.

#### 2.5.5. Cytokine Concentrations Assay

Cell grouping and experimental treatments were conducted as described in Section 2.5.3. After treatment, cell culture supernatants were collected, and the levels of TNF-α and IL-6 were measured using commercial ELISA kits according to the manufacturer’s instructions.

#### 2.5.6. RNA Extraction and Real-Time PCR

Cell grouping and experimental treatments were performed as described in Section 2.5.3. After treatment, cells were harvested for further analysis. Total RNA was extracted using TRIzol reagent and quantified with a NanoDrop 2000 system. Reverse transcription and real-time PCR were performed in strict accordance with the manufacturers’ protocols using the designated kits on an ABI PCR system. The thermal cycling conditions were set as follows: initial denaturation at 95 °C for 3 min; 39 cycles of denaturation at 95 °C for 10 s and annealing/extension at 60 °C for 30 s; followed by a melt curve stage with incubation at 95 °C for 15 s and 63 °C for 30 s, during which fluorescence was measured at 0.05 °C increments from 55 °C to 95 °C. The mRNA expression levels of GSDMD, Casp1, IL-1β, and COX-2 were analyzed using the 2^−ΔΔCt^ method, with β-actin serving as the internal reference  (Table 1).

**Table 1:** Primers used for quantitative reverse transcription polymerase chain reaction for the analysis of inflammatory gene expressions.

Genes	Gene ID		Primers	Amplicon Size (bp)
IL-1β	NM_008361.4	Forward	5′-TGCCACCTTTTGACAGTGATG-3′	138
		Reverse	5′-TGATGTGCTGCTGCGAGATT-3′	
COX-2	NM_011198.5	Forward	5′-AGAAGCGAGGACCTGGGTTCAC-3′	144
		Reverse	5′-ACACCTCTCCACCGATGACCTG-5′	
GSDMD-1	XM_006521343.5	Forward	5′-CGGGCTGAAGCTTTACGGT-3′	60
		Reverse	5′CGACCAAGAGCGGAACTCAG-3′	
Casp1	NM_009807.2	Forward	5′-ACTGCTATGGACAAGGCACG-3′	110
		Reverse	5′-CCTGCCAGGTAGCAGTCTTC-5′	
GAPDH	NM_001289726.2	Forward	5′-GCCTCCTCCAATTCAACCCT-3′	125
		Reverse	5′-TCACACCGACCTTCACCATT-3′	

### 2.6. Animal Experiment

#### 2.6.1. Experimental Design

Fifty male ICR mice (4 weeks of age, weighing 18–22 g) were kept under a controlled environment with a temperature of 23 ± 2 °C, humidity of 50 ± 10%, and a 12/12 h dark/light cycle. They fed normal mouse chow and water ad libitum. After 5 days of acclimatization, mice were divided into 5 groups randomly: the blank control group, the model group, and three E-GDF treatment groups, with 10 mice in each group. Mice in E-GDF treatment groups were gavaged with 50, 100, and 200 mg/kg of the E-GDF once daily for consecutive 7 days, respectively, while mice in the blank and the model control group received an equivalent volume of saline. Throughout the experiment, mice were observed closely for body weight, food and water intake, fur color and sheen, signs of congestion, mental status, and mortality. All experimental procedures were approved by the National Standard Guidelines for Ethical Review of Animal Welfare (GB/T 35892-2018).

After 2 h of administration on day 7, mice in the E-GDF groups and the model group were injected intraperitoneally with 5 mg/kg of LPS, and the blank control group was injected intraperitoneally with an equal amount of saline. After 6 h of intraperitoneal injection of LPS, sampling was performed, and the animals were anesthetized by intraperitoneal injection of tribromoethanol (300 mg/kg, Sigma-Aldrich, St. Louis, MO, USA). After quickly removing the eyeballs, blood was collected into anticoagulant-free tubes, allowed to clot for 30 min at room temperature, then centrifuged at 3000 rpm for 10 min at 4 °C. Serum was collected and stored at −80 °C until analysis. The abdominal cavity was then opened, and the liver was rapidly resected and weighed after removing the fat. A portion of the liver was rinsed with PBS and fixed in 4% paraformaldehyde for histological processing, and the remainder was used to prepare tissue homogenates. The liver tissue was homogenised (1 g liver in 9 mL ice-cold saline) on ice to yield 10% (*w*/*v*) homogenates, which were then centrifuged at 5000 rpm for 10 min at 4 °C, and the supernatants were collected for further analyses.

#### 2.6.2. Liver Index

The final body weights of the mice and the weights of the livers were measured, and the liver index was calculated according to the following formula:

(6) $$Liver\;index\;(\%)=\frac{liver\;mass}{mouse\;body\;weight}\times 100\%$$

#### 2.6.3. Biochemical Analysis and Cytokine Concentrations Assay of Serum

Serum alanine aminotransferase (ALT) and aspartate aminotransferase (AST) levels were measured using Jasco International Co., Ltd. (Tokyo, Japan) with commercially available assay kits, following the manufacturer’s instructions, to assess liver damage. TNF-α and IL-6 were detected in serum using an enzyme-linked immunosorbent assay kit according to the manufacturer’s instructions (Quanzhou Ruixin Biotechnology Co., Ltd., Quanzhou, China) and analyzed using a Multiskan FC enzyme labeler (Thermo Fisher Scientific, Inc., Waltham, MA, USA).

#### 2.6.4. Cytokine Concentrations Assay of Liver

TNF-α and IL-6 levels in the liver homogenate were determined using the same ELISA protocol described above.

#### 2.6.5. Histopathology Assay

Small pieces of liver tissue were fixed in 4% paraformaldehyde for 24 h, dehydrated through graded ethanol, embedded in paraffin wax, and cut into sections of 4 μm thickness.

Sections were stained with hematoxylin and eosin and were digitized at 400× using a VS200 whole-slide scanner (Evident Corporation, Tokyo, Japan). Histopathological damage of the liver was evaluated from the acquired images. Analyzed pathologically under a microscope.

### 2.7. Data Analysis

Experimental data were expressed as mean ± SD. Statistical analyses were carried out with GraphPad Prism 9.0 software (GraphPad Software LLC, San Diego, CA, USA), and comparisons between the two groups were performed using one-way ANOVA. Differences were considered statistically significant when *p* < 0.05.

## 3. Results

### 3.1. Polyphenol and Flavonoid Content of E-GDF

Dried plant powder (200 g) afforded 12.2 g of crude extract (6.1% yield), which was used in all subsequent assays.

Hanh et al. [24] studied the chemical composition of *Leontopodium leontopodioid* (*L. leontopodioid*), which is in the same family of Asteraceae as *G*. *delavayi*, and showed that the main chemical substances of *L. leontopodioid* include flavonoids and polyphenols. In this study, the total phenolic content of the extract was determined using the Folin–Ciocalteu method with gallic acid as a control, and the total flavonoid content was determined using a colorimetric method with rutin as a control. And the results showed that the extract contained 36.15 ± 0.20 mg GAE/g of total phenols and 16.35 ± 0.19 mg RT/g of total flavonoids  (Table 2).

**Table 2:** Total phenolic and flavonoid content of E-GDF.

Plant Extract	Total Polyphenols Content (mg GAE/g d.w. Plant Material)	Total Flavonoids Content (mg RT/g d.w. Plant Material)
E-GDF	36.15 ± 0.20	16.35 ± 0.19

### 3.2. Results of LC-MS Analysis of E-GDF

Non-targeted metabolite detection in positive- and negative-ion modes was carried out by LC-MS, and it was found that E-GDF contained a large number of flavonoids and polyphenols, so the flavonoids and polyphenols were enumerated according to each of the two positive and negative ion modes as the constituents that contained more of them. As shown in Table 3 and Table 4, more than 30 flavonoid constituents were detected in the negative ion mode, including Kaempferol-3-O-rutinoside, Luteolin 7-glucoside, Luteolin, Datiscetin-3-O-rutinoside, Isosakuranin, 5-Demethylnobiletin, Apigenin, Rhoifolin, Apigenin-6-C-glucoside-7-O-glucoside, and 8-Prenylnaringenin. And Phenols compounds included, 3-Hydroxyphenylacetic acid, Hydroxy Ferulic Acid, o-Cresol, Sinapyl alcohol, p-cresol, and 4-Hydroxy-2′,4′,6′-trimethoxychalcone. A total of more than 40 flavonoids were detected in the positive ion mode, of which ten in descending order of content were Luteolin-4′-O-glucoside, Kaempferol-7-O-beta-D-glucopyranoside, Kaempferol-3-O-rutinoside, Luteolin, Karanjin, Glabranine, Apigenin-7-O-glucoside, Quercetin 3-O-alpha-rhamnopyranoside, Rhoifolin, Vitexin. And phenolics including Chlorogenic Acid, 4,5-Dicaffeoylquinic acid, 1,2,3-trihydroxybenzene, Homovanillic acid, Phloroglucinol, Aloe-emodin, 6-Gingerol, 4-Hexylresorcinol, Caffeic Acid, and 3-Hydroxyphenylacetic acid. This is similar to the results of previous compositional analyses of extracts from Asteraceae plants [25,,26,27,28]. It indicates that the E-GDF contains abundant flavonoid and phenolic components, which may possess good anti-inflammatory and antioxidant activities, providing a certain material basis for anti-inflammatory and antioxidant effects in both in vitro and in vivo studies.

**Table 3:** Flavonoid and polyphenol composition of E-GDF in negative ion mode.

Class	Title	Area	Formula	Superclass
Flavonoids	Kaempferol-3-O-rutinoside	1,024,976.0	C_27_H_30_O_15_	Phenylpropanoids and polyketides
Flavonoids	Luteolin 7-glucoside	784,785.1	C_21_H_20_O_11_	Phenylpropanoids and polyketides
Flavonoids	Luteolin	205,475.8	C_15_H_10_O_6_	Phenylpropanoids and polyketides
Flavonoids	Datiscetin-3-O-rutinoside	194,335.5	C_27_H_30_O_15_	Phenylpropanoids and polyketides
Flavonoids	Isosakuranin	163,102.3	C_22_H_24_O_10_	Phenylpropanoids and polyketides
Flavonoids	5-Demethylnobiletin	145,882.1	C_20_H_20_O_8_	Phenylpropanoids and polyketides
Flavonoids	Apigenin	120,222.9	C_15_H_10_O_5_	Phenylpropanoids and polyketides
Flavonoids	Rhoifolin	109,303.2	C_27_H_30_O_14_	Phenylpropanoids and polyketides
Flavonoids	Apigenin-6-C-glucoside-7-O-glucoside	91,202.4	C_27_H_30_O_15_	Phenylpropanoids and polyketides
Flavonoids	8-Prenylnaringenin	83,827.2	C_20_H_20_O_5_	Phenylpropanoids and polyketides
Phenols	3-Hydroxyphenylacetic acid	732,799.1	C_8_H_8_O_3_	Benzenoids
Phenols	Hydroxy Ferulic Acid	398,804.3	C_10_H_10_O_5_	Phenylpropanoids and polyketides
Phenols	o-Cresol	78,590.8	C_7_H_8_O	Benzenoids
Phenols	Sinapyl alcohol	77,982.2	C_11_H_14_O_4_	Benzenoids
Phenols	p-cresol	60,442.5	C_7_H_8_O	Benzenoids
Phenols	4-Hydroxy-2′,4′,6′-trimethoxychalcone	40,385.5	C_18_H_18_O_5_	Phenylpropanoids and polyketides
Phenols	4-Hydroxy-3-methoxymandelate	25,971.8	C_9_H_10_O_5_	Benzenoids

**Table 4:** Flavonoid and polyphenol composition of E-GDF in positive ion mode.

Class	Title	Area	Formula	Superclass
Flavonoids	Luteolin-4′-O-glucoside	1,498,349.9	C_21_H_20_O_11_	Phenylpropanoids and polyketides
Flavonoids	Kaempferol-7-O-beta-D-glucopyranoside	1,429,160.9	C_21_H_20_O_11_	Phenylpropanoids and polyketides
Flavonoids	Kaempferol-3-O-rutinoside	861,844.4	C_27_H_30_O_15_	Phenylpropanoids and polyketides
Flavonoids	Luteolin	834,777.2	C_15_H_10_O_6_	Phenylpropanoids and polyketides
Flavonoids	Karanjin	562,061.5	C_18_H_12_O_4_	Phenylpropanoids and polyketides
Flavonoids	Glabranine	426,142.6	C_20_H_20_O_4_	Phenylpropanoids and polyketides
Flavonoids	Apigenin-7-O-glucoside	353,608.5	C_21_H_20_O_10_	Phenylpropanoids and polyketides
Flavonoids	Quercetin 3-O-alpha-rhamnopyranoside	249,107.1	C_21_H_20_O_11_	Phenylpropanoids and polyketides
Flavonoids	Rhoifolin	217,541.6	C_27_H_30_O_14_	Phenylpropanoids and polyketides
Flavonoids	Vitexin	152,100.1	C_21_H_20_O_10_	Phenylpropanoids and polyketides
Phenylpropanoids	Chlorogenic Acid	2,349,359.8	C_16_H_18_O_9_	Polyphenols
Phenylpropanoids	4,5-Dicaffeoylquinic acid	1,518,505.8	C_25_H_24_O_12_	Polyphenols
Phenols	1,2,3-trihydroxybenzene	444,096.6	C_6_H_6_O_3_	Benzenoids
Phenols	Homovanillic acid	205,465.3	C_9_H_10_O_4_	Benzenoids
Phenols	Phloroglucinol	113,469.5	C_6_H_6_O_3_	Benzenoids
Phenols	Aloe-emodin	85,216.2	C_15_H_10_O_5_	Benzenoids
Phenols	6-Gingerol	65,219.4	C_17_H_26_O_4_	Benzenoids
Phenols	4-Hexylresorcinol	58,185.9	C_12_H_18_O_2_	Benzenoids
Phenolic acids	Caffeic Acid	56,376.8	C_9_H_8_O_4_	Hydroxycinnamic acids
Phenols	3-Hydroxyphenylacetic acid	30,506.9	C_8_H_8_O_3_	Benzenoids

### 3.3. In Vitro Antioxidant Results

E-GDF is rich in polyphenols and flavonoids, which are hypothesized to have a certain antioxidant effect. In this experiment, using ascorbic acid as the control, the scavenging effect of E-GDF on the DPPH radical, ABTS, ^•^OH radical, and O_2_^•−^ radical was assessed. The results are shown in Table 5, it shows that the ability of the extract to scavenge DPPH, ^•^OH, and O_2_^•−^ is significantly lower than that of reference substances, and the extract’s ability to scavenge the ABTS free radicals is ability was comparable to that of the reference substance. The IC_50_ values of E-GDF against the DPPH radical, ABTS radical, ^•^OH radical, and O_2_^•−^ radical were 12.9 mg/mL, 8.0 mg/mL, 3.2 mg/mL, and 15.7 mg/mL, respectively. These results suggested that E-GDF has a certain antioxidant capacity.

**Table 5:** The capacity of E-GDF to scavenge free radicals.

	Inhibition Rate (%)				EC_50_ (mg/mL)			
	DPPH	ABTS	•OH	O_2_^•−^	DPPH	ABTS	•OH	O_2_^•−^
Vitamin C (control)	96.27± 0.01	73.62 ± 0.01	99.87 ± 0.01	98.89 ± 0.01	-	-	-	-
E-GDF	62.32 ± 0.02	72.66 ± 0.01	70.70 ± 0.03	60.82± 0.04	12.9	8.0	3.2	15.7

### 3.4. RAW264.7 Cell Experiments

#### 3.4.1. Determination of Cell Viability by CCK-8 Method

**Figure 1:**
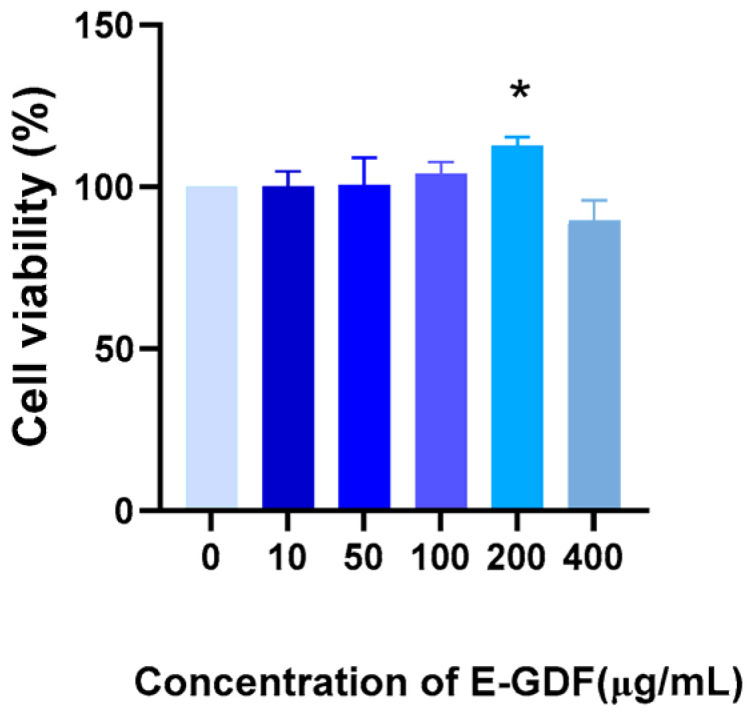
Cell viability was assessed using CCK-8 assay after treatment with E-GDF (0, 10, 50, 100, 200, and 400 μg/mL). *n* = 3. * *p* < 0.05, compared with the blank control group.

The effects of serial concentrations of E-GDF (0, 10, 50, 100, 200, 400 μg/mL) on cell viability were determined using CCK-8 assay. The results, as presented in Figure 1, showed that E-GDF was not cytotoxic to RAW264.7 cells at concentrations ranging from 10 to 400 μg/mL. A slight decrease in cell viability was observed only at the highest concentration (400 μg/mL). Therefore, E-GDF doses between 10 and 200 μg/mL were considered safe, and the concentrations of 50, 100, and 200 μg/mL were selected as the low, medium, and high dose concentrations for subsequent cell experiments.

#### 3.4.2. Determination of Intracellular Reactive Oxygen Species (ROS) Levels

**Figure 2:**
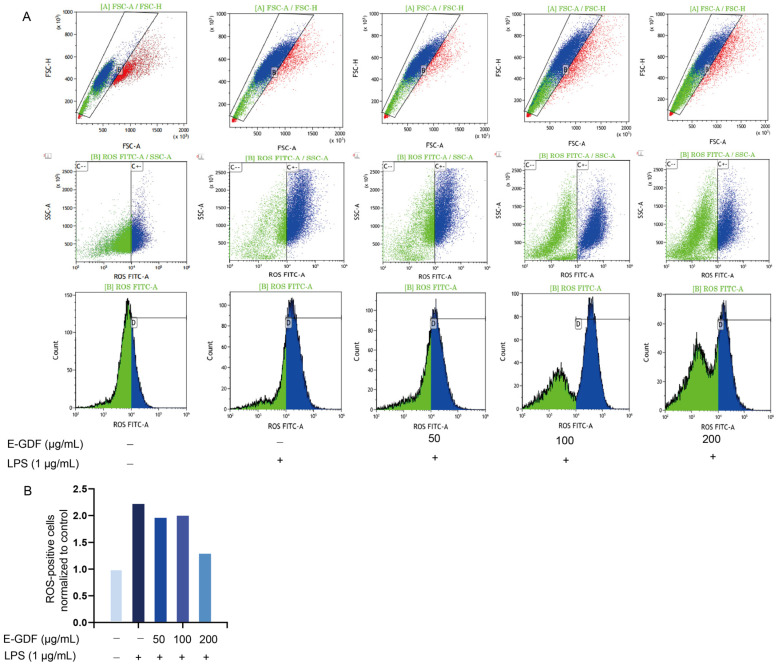
Flow cytometry analysis of LPS-challenged RAW264.7 cells treated with E-GDF. (**A**) There are five columns in the figure, and each column represents an experimental group, from left to right: blank group, model group, low-dose group, medium-dose group, and high-dose group. (**B**) The proportions of ROS-positive cells in each group, with the control group normalized as 100%.

LPS-induced ROS production in RAW264.7 cells was analyzed by flow cytometry after treatment with graded concentrations of E-GDF, and the results are shown in the histogram in Figure 2A,B. The proportion of ROS-positive cells in the blank control group was 30.54%. Following LPS stimulation, the fluorescence peak of the model group shifted significantly to the right compared with that of the blank control group, and the proportion of ROS-positive cells increased dramatically to 72.5%, confirming the successful establishment of the oxidative-stress model. However, treatment with different concentrations of E-GDF abolished LPS-induced ROS production, and the proportion of ROS-positive cells was reduced to 61.24%, 61.22%, and 40.63% in the low, medium, and high dose groups, respectively.

#### 3.4.3. Inflammatory Factor Levels

**Figure 3:**
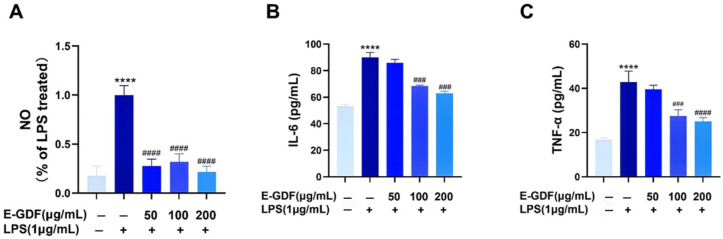
Effects of E-GDF on the production of inflammatory factors in LPS-challenged RAW264.7 cells. (**A**) NO content; (**B**) IL-6 levels; (**C**) IL-1β levels. Statistical analyses were performed according to one-way ANOVA and multiple comparison tests. All data were expressed as mean ± SD of three independent experiments. **** *p* < 0.0001, compared with the blank control group; ### *p* < 0.001, #### *p* < 0.0001, compared with the model group.

To assess the anti-inflammatory potential of E-GDF, the levels of NO, IL-6, and TNF-α in RAW264.7 cells were determined, and the results are shown in Figure 3. Compared with the blank control group, the model group showed a highly significant increase in NO, IL-6, and TNF-α levels in LPS-challenged RAW264.7 cells. However, the E-GDF groups significantly reduced the LPS-induced NO production(*p* < 0.0001) (Figure 3A). Figure 3B,C shows that E-GDF treatment significantly decreased the LPS-induced IL-6 and TNF-α levels in RAW264.7 cells at concentrations of 100 and 200 μg/mL (*p* < 0.001).

#### 3.4.4. The mRNA Expression of Pro-Inflammatory Mediators

**Figure 4:**
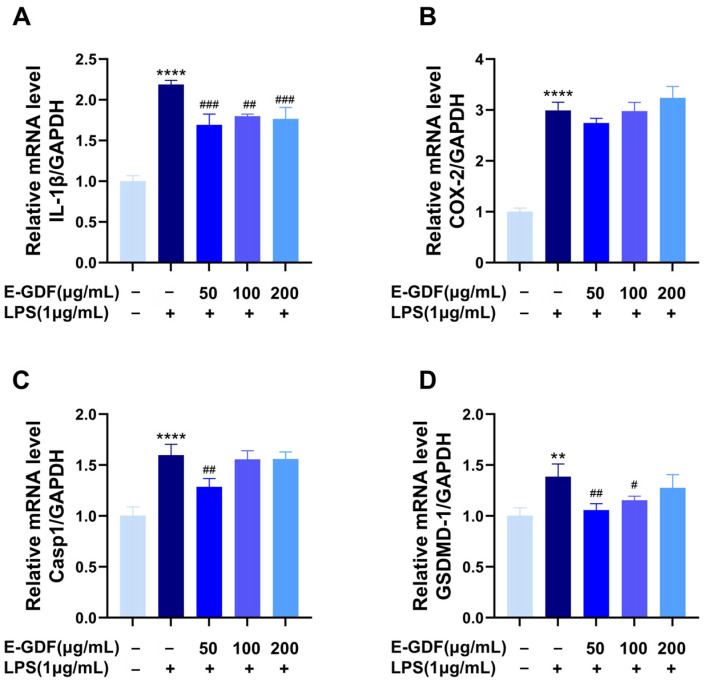
Effects of E-GDF on mRNA levels of inflammatory factors in LPS-challenged RAW264.7 cells. (**A**) IL-1β; (**B**) COX-2; (**C**) Casp1; (**D**) GSDMD-1. Statistical analyses were performed according to one-way ANOVA and multiple comparison tests. All data are expressed as mean ± SD of three independent experiments. ** *p* < 0.01, **** *p* < 0.0001, compared with the blank control group; # *p* < 0.05, ## *p* < 0.01, ### *p* < 0.001, compared with the model group.

In the experiment, RT-qPCR was used to measure the relative mRNA expression levels of proinflammatory mediators. Results are shown in Figure 4, compared with the blank control group, LPS stimulation significantly increased the mRNA expression of IL-1β, COX-2, Casp1, and GSDMD-1 in the model group. Compared with the model group, various concentrations of E-GDF significantly reduced LPS-induced IL-1β mRNA expression (Figure 4A). At 50 μg/mL, E-GDF significantly decreased LPS-induced Casp1 and GSDMD-1 mRNA expression (Figure 4C,D), while 100 μg/mL E-GDF significantly reduced LPS-induced GSDMD-1 mRNA expression (Figure 4D). However, E-GDF at all tested concentrations had no significant effect on COX-2 mRNA expression (Figure 4B).

### 3.5. Animal Experiments

#### 3.5.1. Effect of E-GDF on Liver Index of Mice Induced by LPS

**Figure 5:**
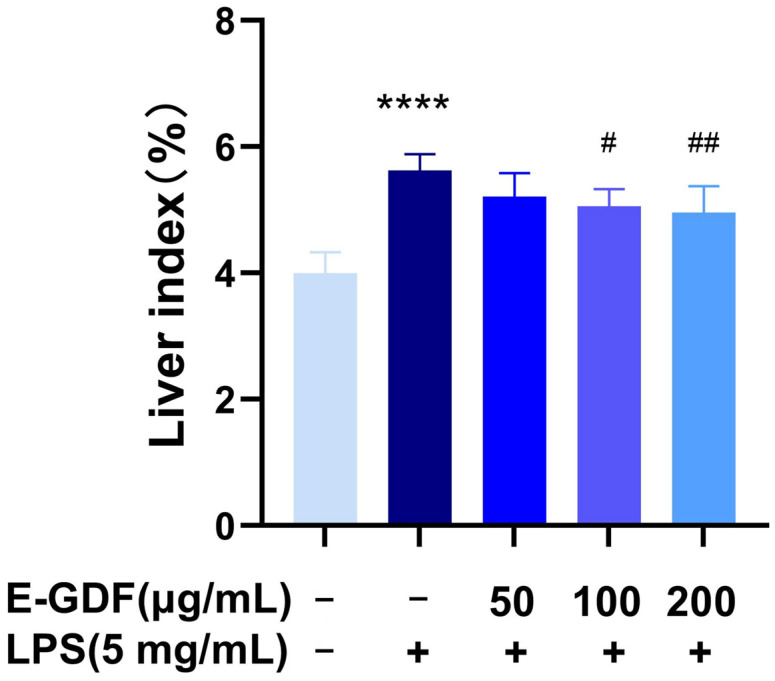
Effect of E-GDF on liver index (Ratio of liver weight to body weight) in LPS-induced septic mice. Statistical analyses were performed according to one-way ANOVA and multiple comparison test. All data are expressed as mean ± SD of three independent experiments. **** *p* < 0.0001 compared with the blank control group; # *p* < 0.05 and ## *p* < 0.01 compared with the model group.

The effects of E-GDF on LPS-induced liver index in mice are shown in Figure 5. Compared with the blank group, the liver index was significantly elevated in the model group. Following E-GDF treatment, the liver index exhibited a dose-dependent decrease. Compared with the model group, the low-dose group (50 mg/kg) showed no significant difference, whereas significant reductions were observed in both the medium-dose (100 mg/kg) and high-dose (200 mg/kg) groups.

#### 3.5.2. Serum AST and ALT Levels

**Figure 6:**
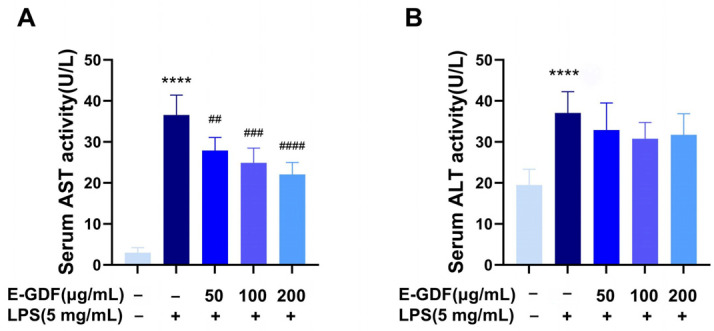
Effect of E-GDF on LPS induced serum ALT and AST levels. (**A**) AST viability in mouse serum; (**B**) ALT viability in mouse serum. Statistical analysis based on one-way ANOVA and multiple comparison test. All data are expressed as mean ± SD of three independent experiments. **** *p* < 0.0001, compared with the blank control group; ## *p* < 0.01, ### *p* < 0.001, #### *p* < 0.0001, compared with the model group.

Figure 6 shows the effects of E-GDF on LPS-induced serum ALT and AST levels. Compared with the blank group, serum AST and ALT activities were significantly elevated in the model group mice. However, E-GDF administration significantly reduced AST activity in a dose-dependent manner (Figure 6A). There was also a trend towards decreased serum ALT in E-GDF treatment groups (Figure 6B).

#### 3.5.3. Effect of E-GDF on LPS-Induced Pro-Inflammatory Cytokine Production

**Figure 7:**
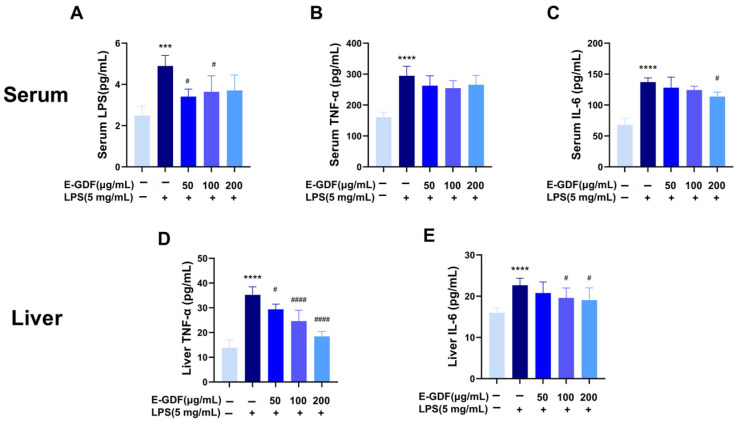
Effects of E-GDF on LPS-induced inflammatory factors production of serum and liver in mice. (**A**–**C**) Serum LPS, TNF-α, and IL-6 levels; (**D**,**E**) liver TNF-α and IL-6 levels. Statistical analyses were performed according to one-way ANOVA and multiple comparison tests. All data are expressed as mean ± SD of three independent experiments. **** *p* < 0.0001, *** *p* < 0.001 compared with the blank control group; # *p* < 0.05, #### *p* < 0.0001, compared with the model group.

LPS-stimulated macrophages secrete pro-inflammatory cytokines, such as TNF-α and IL-6, which are required for inflammatory diseases. To investigate the anti-inflammatory effects of E-GDF in vivo models, mouse models of liver inflammation were induced by LPS and treated with E-GDF. Serum LPS content, TNF-α, and IL-6 levels, as well as liver TNF-α and IL-6 levels, were determined. As shown in Figure 7, compared with the blank control group, serum LPS, IL-6, and TNF-α levels, as well as hepatic TNF-α and IL-6 levels, significantly increased upon LPS challenge (*p* < 0.0001) in the model group. In contrast, E-GDF treatment significantly reduced serum LPS content at the concentration of 50 μg/mL (*p* < 0.05, Figure 7A) as well as IL-6 levels at the concentration of 200 μg/mL (Figure 7C) (*p* < 0.05). But there was no significant change in serum TNF-α. Meanwhile, E-GDF treatment significantly reduced hepatic TNF-α and IL-6 levels in a dose-dependent manner (Figure 7D,E).

#### 3.5.4. Liver Histopathological Sections

**Figure 8:**
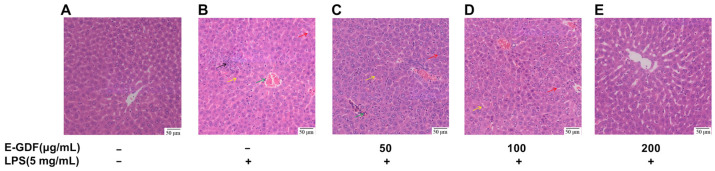
Histopathological analysis of the mouse liver. Red arrow: hepatocyte enlargement; yellow arrow: hepatocyte necrosis; black arrow: necrotic foci; green arrow: inflammatory cells. All sections were stained with H&E, and the magnification is 200×.

At the end of the experiment, liver tissues were collected and subjected to histopathological analysis. Detailed histopathological changes in the livers of all experimental groups are presented in Figure 8. The liver structure remained intact in the blank control group. Hepatocytes were neatly arranged, hepatic sinusoids were clearly visible, hepatic cords were orderly aligned, and nuclear morphology was normal. LPS treatment of mouse livers exhibited disorganized hepatocyte and hepatic cord arrangement, hepatocyte swelling and necrosis, necrotic foci, and inflammatory cell infiltration. E-GDF treatment revealed a dose-dependent histological improvement, with all three dose groups exhibiting markedly less liver injury than the model group. Especially in the high-dose group (200 mg/kg). Liver architecture was relatively intact without signs of hepatocyte necrosis, haemorrhage, or inflammation.

## 4. Discussion

*G*. *delavayi*, a species of the genus Gerbera in the Asteraceae family, is currently used as an ethnic medicine in the Yi ethnic region of China. To date, research on the phytochemical composition and pharmacological activity of *G*. *delavayi* remains extremely limited. In our study, we first conducted a preliminary study on the polyphenolic components of *G*. *delavayi*. Flavonoids, phenolic acids, and other polyphenolic compounds are regarded as important components in the *Gerbera genus* of the Asteraceae family [29]. In the present research, our quantitative colorimetric assays revealed that E-GDF was rich in polyphenols (36.15 ± 0.20 mg GAE/g) and flavonoids (16.35 ± 0.19 mg RT/g). Asen’s study also revealed that Gerbera plants are rich in flavonoids [30]. Flavonoids and phenolic acid components in E-GDF were further analyzed by LC-MS. More than 40 flavonoid compounds were identified, among which kaempferol glycosides, luteolin, and their glycosidic derivatives predominated. The phenolic acids in the extract were dominated by chlorogenic, caffeic, and ferulic acids together with their respective glycosides. Flavonoids and phenolic acids are generally recognized for their excellent antioxidant capacity; consequently, we evaluated the antioxidant activity of E-GDF in this study. The results showed that the extract exhibited great scavenging activity against DPPH, ABTS, ^•^OH, and O_2_^•−^ radicals in vitro. This is similar to the strong in vitro antioxidant activity observed in *L. leontopodioides*, another Asteraceae plant rich in chlorogenic and ferulic acids [31,32,33].

The antioxidant capacity of E-GDF was further corroborated at the cellular level. Flow-cytometric analysis demonstrated that E-GDF markedly attenuated LPS-elicited ROS production in RAW264.7 macrophages, underscoring its potent intracellular antioxidant activity. The abundant flavonoids and phenolic compounds in E-GDF may be the material basis for its in vitro antioxidant activity.

Ethanolic plant extracts rich in phenolic acids have also been shown to have excellent anti-inflammatory activity [34]. The LPS-induced RAW264.7 macrophage inflammation model is commonly used to assess the anti-inflammatory activity of plant extracts [35]. In this experiment, LPS-challenged RAW264.7 cells were activated and released a large amount of inflammatory mediators such as NO, IL-6, and TNF-α. E-GDF significantly inhibited NO secretion and reduced the production of pro-inflammatory mediators (IL-6 and TNF-α). Moreover, E-GDF also downregulated COX-2 and IL-1β mRNA expression. These results all suggested that E-GDF possesses good in vitro anti-inflammatory activity. Extracts from other Asteraceae plants, such as *L*. *leontopodioides* and *G*. *piloselloides*, have likewise been shown to exert pronounced anti-inflammatory effects. Cell experiments also confirmed that E-GDF can downregulate the mRNA expression levels of Casp1, GSDMD-1, IL-1β, and IL-6. Caspase-1 and GSDMD are the pivotal executors of pyroptosis, and the pyroptotic process mediated by them is often accompanied by a strong inflammatory response. Its activation invariably elicits a pro-inflammatory signature, of which IL-1β secretion constitutes a canonical hallmark [36]. In the study, our findings suggest that E-GDF may, at least in part, exert anti-inflammatory effects through down-regulating pyroptosis.

The liver serves as a critical defense against microbial invasion and is also a primary target of inflammatory imbalance. Acute liver injury is a common complication of sepsis induced by high doses of LPS [37,38]. Numerous studies have demonstrated that flavonoids [39] and polyphenolic [40] compounds exhibit protective effects against septic liver injury. In this study, we investigated the protective effects of E-GDF on LPS-induced acute liver injury [41]. Based on a preliminary dose-range finding study (10, 22, 50, 10, 200 mg/kg), doses of 50, 100, and 200 mg/kg were selected, which were well-tolerated and showed preliminary hepatoprotective effects. Abnormal liver index partly reflects the severity of hepatomegaly and facilitates evaluation of the hepatic pathological state [42], whereas serum ALT and AST are established biochemical hallmarks of hepatocellular injury. Our results demonstrate that E-GDF significantly reversed LPS-induced increases in liver index and reduced serum AST levels, while ALT exhibited a downward trend, indicating its potential hepatoprotective effects. Liver histopathological changes remain the gold standard for evaluating the severity of hepatic injury. Compared with the model group, E-GDF treatment reduced liver injury in mice in a clear dose-dependent manner. Especially the high-dose group, showed markedly attenuated hepatocellular necrosis and reduced inflammatory infiltration, with hepatic architecture closely resembling that of the normal group. These results further confirm that E-GDF effectively counteracts LPS-induced structural liver damage. Liver injury in sepsis is frequently accompanied by severe hepatic inflammation. In this experiment, after high-dose LPS-induced sepsis in mice, serum LPS concentration and TNF-α/IL-6 levels in both serum and liver increased significantly, denoting profound systemic inflammation with concomitant severe hepatic inflammatory injury. E-GDF administration significantly lowered serum LPS concentration and suppressed IL-6 and TNF-α levels in both circulation and liver tissue, demonstrating that it protects the liver by blocking endotoxin and these pivotal inflammatory mediators.

## 5. Conclusions

Ethanolic extract of *G. delavayi* is abundant in flavonoids such as kaempferol and luteolin, as well as phenolic acids including chlorogenic acid, caffeic acid, and ferulic acid. This endows the extract with potential antioxidant and anti-inflammatory activities. Additionally, the *G. delavayi* extract demonstrates good protective effects against LPS-induced sepsis-related acute liver injury, which may be related to its anti-pyroptosis activity. These findings suggest the therapeutic potential of *G. delavayi* extract for anti-inflammatory and hepatoprotective applications. However, the major active constituents responsible for the observed effects remain uncharacterized, and the precise mechanisms underlying its hepatoprotective action are not fully elucidated. Future studies are warranted to isolate and identify the key bioactive compounds and to delineate their molecular targets and pathways.

## Data Availability

The original contributions presented in the study are included in the article; further inquiries can be directed to the corresponding author.

## References

[1] Ferrero-Miliani L., Nielsen O.H., Andersen P.S., Girardin S.E. (2007). Chronic inflammation: Importance of NOD2 and NALP3 in interleukin-1beta generation. Clin. Exp. Immunol..

[2] Kim K.M., Kim S.Y., Mony T.J., Bae H.J., Han S.D., Lee E.S., Choi S.H., Hong S.H., Lee S.D., Park S.J. (2021). Dracocephalum moldavica Ethanol Extract Suppresses LPS-Induced Inflammatory Responses through Inhibition of the JNK/ERK/NF-κB Signaling Pathway and IL-6 Production in RAW 264.7 Macrophages and in Endotoxic-Treated Mice. Nutrients.

[3] Andrade R.J., Chalasani N., Björnsson E.S., Suzuki A., Kullak-Ublick G.A., Watkins P.B., Devarbhavi H., Merz M., Lucena M.I., Kaplowitz N. (2019). Drug-induced liver injury. Nat. Rev. Dis. Primers.

[4] Seo H.Y., Park J.Y., Lee S.H., Cho S.H., Han E., Hwang J.S., Kim M.K., Jang B.K. (2025). Clusterin inhibits lipopolysaccharide induced liver injury. Sci. Rep..

[5] Xu D., Xu M., Jeong S., Qian Y., Wu H., Xia Q., Kong X. (2018). The Role of Nrf2 in Liver Disease: Novel Molecular Mechanisms and Therapeutic Approaches. Front. Pharmacol..

[6] Qi C., Li L., Cheng G., Xiao B., Xing Y., Zhao X., Liu J. (2021). Platycodon grandiflorus Polysaccharide with Anti-Apoptosis, Anti-Oxidant and Anti-Inflammatory Activity Against LPS/D-GalN Induced Acute Liver Injury in Mice. J. Polym. Environ..

[7] Robinson N., Ganesan R., Hegedűs C., Kovács K., Kufer T.A., Virág L. (2019). Programmed necrotic cell death of macrophages: Focus on pyroptosis, necroptosis, and parthanatos. Redox Biol..

[8] Lee S.C., Ahn J., Kim J., Lee J.Y., Kim J., Uddin M.S., Lee S.W., Kim C.Y. (2023). The Antioxidant and Anti-Inflammatory Properties of Merremia umbellata Extract. Antioxidants.

[9] Yang Y.X., Wang Q., Huang H.Y., Wang Z.J. (2023). New 5-methyl-4-hydroxycoumarin polyketide derivatives from Gerbera delavayi with anti-inflammatory activity. Fitoterapia.

[10] Pietiäinen M., Kontturi J., Paasela T., Deng X., Ainasoja M., Nyberg P., Hotti H., Teeri T.H. (2016). Two polyketide synthases are necessary for 4-hydroxy-5-methylcoumarin biosynthesis in Gerbera hybrida. Plant J. Cell Mol. Biol..

[11] Zhao C., Gao H., Li J., Yu M., Wu J., Zhang H., Zhang T., Zou Z. (2022). Bioactive constituents from Gerbera piloselloides with anti-inflammatory and antiproliferative activities. Fitoterapia.

[12] Chen S.L., Gao H., Zhao C.X., Zhang T., Zou Z.M. (2025). LC-MS coupled with diagnostic ion strategy facilitated the discovery of 5-methylcoumarin meroterpenoids from Gerbera piloselloides. Phytochemistry.

[13] Ullah M.S., Amjad A., Chauhdary Z., Saleem U., Akhtar N. (2025). Phytochemical studies of Gerbera jamesonii and evaluation of anti-inflammatory potential in formaldehyde-induced arthritis in rats. Inflammopharmacology.

[14] Liu C., Fu C., Lu Y., Sun J., Liu T., Wang Y., Wang A., Huang Y., Li Y. (2024). Integration of metabolomics and transcriptomics to reveal the mechanism of *Gerberae piloselloidis* herba in alleviating bronchial asthma. J. Ethnopharmacol..

[15] Zhou K., Lu D., You J., Liu T., Sun J., Lu Y., Pan J., Li Y., Liu C. (2022). Integrated plasma pharmacochemistry and network pharmacology to explore the mechanism of *Gerberae piloselloidis* Herba in treatment of allergic asthma. J. Ethnopharmacol..

[16] Zhao C., Li J., Hu Y., Li L., Yu M., Huang Y., Zhang T., Shang H., Zou Z. (2024). (+)/(−)-Gerbeloid A, a pair of unprecedented coumarin-based polycyclic meroterpenoid enantiomers from *Gerbera piloselloides*: Structural elucidation, semi-synthesis, and lipid-lowering activity. Acta Pharm. Sin. B.

[17] He F., Yang J., Cheng X., Wang R., Qu H., Jiang H., Bai Y., Cao W. (2019). 8-methoxysmyrindiol from *Gerbera piloselloides* (L.) Cass. and its vasodilation effects on isolated rat mesenteric arteries. Fitoterapia.

[18] He F., Wang M., Gao M., Zhao M., Bai Y., Zhao C. (2014). Chemical Composition and Biological Activities of *Gerbera anandria*. Molecules.

[19] Chera E.I., Pop R.M., Pârvu M., Sorițău O., Uifălean A., Cătoi F.A., Cecan A., Negoescu A.G., Achimaș-Cadariu P., Pârvu A.E. (2022). Flaxseed Ethanol Extracts’ Antitumor, Antioxidant, and Anti-Inflammatory Potential. Antioxidants.

[20] Macías-Cortés E., Gallegos-Infante J.A., Rocha-Guzmán N.E., Moreno-Jiménez M.R., Cervantes-Cardoza V., Castillo-Herrera G.A., González-Laredo R.F. (2022). Antioxidant and anti-inflammatory polyphenols in ultrasound-assisted extracts from salvilla (*Buddleja scordioides* Kunth). Ultrason. Sonochemistry.

[21] Chen H., Zeng J., Wang B., Cheng Z., Xu J., Gao W., Chen K. (2021). Structural characterization and antioxidant activities of *Bletilla striata* polysaccharide extracted by different methods. Carbohydr. Polym..

[22] Deng T., Hu S., Huang X.-A., Song J., Xu Q., Wang Y., Liu F. (2019). A novel strategy for colorimetric detection of hydroxyl radicals based on a modified Griess test. Talanta.

[23] Liu F., Ooi V.E., Chang S.T. (1997). Free radical scavenging activities of mushroom polysaccharide extracts. Life Sci..

[24] Hanh N.T., Ha N.M., Van C.A., Pham T.V., Son N.T. (2025). Leontopodium species: Phytochemistry, biosynthesis, synthesis, pharmacology, and synthetic advancement. Fitoterapia.

[25] Li L., Zhang G.G., Zuo T.T., Wu S.H. (2008). Chemical constituents of *Leontopodium leontopodioid* (wild.) Beauv. Cent. South Pharm..

[26] Chen Q., Li J., Ruan J., Qu L., Wei H., Ma X., Zhang Y., Wang T. (2018). Bioactive constituents from the whole plants of *Leontopodium leontopodioides* (Wild.) Beauv. J. Nat. Med..

[27] Wu Y., Ding C., Zhang Z., Zhang J., Li Y., Song X., Zhang D. (2024). Sesquilignans: Current research and potential prospective. Eur. J. Med. Chem..

[28] Safer S., Cicek S.S., Pieri V., Schwaiger S., Schneider P., Wissemann V., Stuppner H. (2011). Metabolic fingerprinting of *Leontopodium* species (Asteraceae) by means of ^1^H NMR and HPLC-ESI-MS. Phytochemistry.

[29] Olech M., Łyko L., Nowak R. (2020). Influence of Accelerated Solvent Extraction Conditions on the LC-ESI-MS/MS Polyphenolic Profile, Triterpenoid Content, and Antioxidant and Anti-lipoxygenase Activity of *Rhododendron luteum* Sweet Leaves. Antioxidants.

[30] Asen S. (1984). High pressure liquid chromatographic analysis of flavonoid chemical markers in petals from Gerbera flowers as an adjunct for cultivar and germplasm identification. Phytochemistry.

[31] Ramírez-Brewer D., Quintana-Martinez S.E., García-Zapateiro L.A. (2025). Obtaining and characterization of natural extracts from mango (*Mangifera indica*) peel and its effect on the rheological behavior in new mango kernel starch hydrogels. Food Chem..

[32] Shen S., Wang J., Chen X., Liu T., Zhuo Q., Zhang S.Q. (2019). Evaluation of cellular antioxidant components of honeys using UPLC-MS/MS and HPLC-FLD based on the quantitative composition-activity relationship. Food Chem..

[33] Rui L., Xie M., Hu B., Zhou L., Saeeduddin M., Zeng X. (2017). Enhanced solubility and antioxidant activity of chlorogenic acid-chitosan conjugates due to the conjugation of chitosan with chlorogenic acid. Carbohydr. Polym..

[34] Lin J.T., Chang Y.Y., Chen Y.C., Shen B.Y., Yang D.J. (2017). Molecular mechanisms of the effects of the ethanolic extract of *Muntingia calabura* Linn. fruit on lipopolysaccharide-induced pro-inflammatory mediators in macrophages. Food Funct..

[35] Liu H.J., Li J., Xie H.Y., Wang L.L., Zhang Z.Z., Yan C. (2021). Anti-inflammatory effects of diaporisoindole B in LPS-stimulated RAW 264.7 macrophage cells via MyD88 activated NF-κB and MAPKs pathways. J. Chin. Pharm. Sci..

[36] Dinarello C.A. (2018). Overview of the IL-1 family in innate inflammation and acquired immunity. Immunol. Rev..

[37] Zhang B., Fan C., Tan Q., Zhang Y., Jiang Q., Yu Q., Zhang B., Zheng K., Yan C. (2022). rCsHscB Derived from Clonorchis sinensis: A Carcinogenic Liver Fluke Ameliorates LPS-Induced Acute Hepatic Injury by Repression of Inflammation. Pathogens.

[38] Chen S.N., Tan Y., Xiao X.C., Li Q., Wu Q., Peng Y.Y., Ren J., Dong M.L. (2021). Deletion of TLR4 attenuates lipopolysaccharide-induced acute liver injury by inhibiting inflammation and apoptosis. Acta Pharmacol. Sin..

[39] He Y., Xia Z., Yu D., Wang J., Jin L., Huang D., Ye X., Li X., Zhang B. (2019). Hepatoprotective effects and structure-activity relationship of five flavonoids against lipopolysaccharide/d-galactosamine induced acute liver failure in mice. Int. Immunopharmacol..

[40] Xu Y., Chen J., Yu X., Tao W., Jiang F., Yin Z., Liu C. (2010). Protective effects of chlorogenic acid on acute hepatotoxicity induced by lipopolysaccharide in mice. Inflamm. Res..

[41] Strnad P., Tacke F., Koch A., Trautwein C. (2017). Liver—Guardian, modifier and target of sepsis. Nat. Rev. Gastroenterol. Hepatol..

[42] Eid B.G., El-Shitany N.A. (2021). Captopril downregulates expression of Bax/cytochrome C/caspase-3 apoptotic pathway, reduces inflammation, and oxidative stress in cisplatin-induced acute hepatic injury. Biomed Pharmacother..

